# Thymic tumors: radiotherapy experience for single institute

**DOI:** 10.1007/s00066-025-02395-y

**Published:** 2025-04-23

**Authors:** Sureyya Sarihan, Aybuke Tugce Metin, Ahmet Sami Bayram, Huseyin Melek

**Affiliations:** 1 Faculty of Medicine, Department of Radiation Oncology, Bursa Uludag University, 16059 Bursa, Turkey; 2 Faculty of Medicine, Department of Thoracic Surgery, Bursa Uludag University, 16059 Bursa, Turkey

**Keywords:** Thymic tumors, Radiotherapy, Local control, Survival

## Abstract

**Purpose:**

The aim is to evaluate treatment outcomes and prognostic factors in patients with thymic epithelial tumor (TET) treated with radiotherapy (RT).

**Methods:**

Sixty-four patients were treated between 2000 and 2023. The median age was 52 years (20–83), and 81% of underwent R0 resection. The stage (s) distribution for I, II, III, and IV were 5%, 61%, 26%, and 8% by Masaoka-Koga and 63%, 11%, 17%, and 9% by TNM, respectively. WHO types A/AB/B/C and thymic neuroendocrine tumors were seen in 5%, 22%, 64%, 6%, and 3% of patients, respectively. The median RT dose was 5040 cGy (1620–6596). Survival was calculated from the beginning of RT.

**Results:**

The median follow-up was 70 months (1.5–268). The median time to recurrence was 30 months (6.5–106), seen in 23% of patients. Mean overall (OS), progression-free survival (PFS) and 5‑year local control were 141, 138 months, and 82.4%, respectively. In univariate analysis, the presence of organ invasion and TNM stage were significant as new prognostic factors for survival (*p* < 0.05). In multivariate analysis, the high-risk group (B2/B3/C) and another surgical center (*p* < 0.05) for OS, and KPS ≤ 80, thymic carcinoma, and Masaoka-Koga sIII-IV (*p* < 0.05) for PFS were identified as unfavorable prognostic factors.

**Conclusion:**

Recurrence in TET can occur over a longer period. In this study, 5‑year local control of 82.4% was achieved. The prognostic importance of KPS, histology, Masaoka-Koga stage, risk group, and surgical center was demonstrated. Advances in the diagnosis, staging, and treatment of TET will enable more personalized treatment.

## Introduction

Thymic epithelial tumors (TET) are a heterogeneous group including thymomas, thymic carcinomas (TC), and thymic neuroendocrine tumors (TNET) [1]. The incidence for all TET patients is reported as 1.3–3.2 per million. In a recent study, the incidence rates for thymoma, TC and TNET were reported as 88%, 10 and 2%, respectively [2]. The median age at diagnosis is 50–60 years, and they constitute 35–50% of anterior mediastinal tumors [1–3]. Thymomas are further classified according to WHO histopathological criteria into types A, AB, B1, B2, and B3, and rare variants such as atypical type A, micronodular thymoma, and metaplastic thymoma are included in the 2021 classification [4]. In the Réseau Tumeurs Thymiques et Cancer (RYTHMIC) study, the frequency of occurrence for WHO types A, AB, B, C, and others were reported as 10%, 25%, 53%, 11, and 1% respectively [5]. One-third of cases are associated with autoimmune diseases, with myasthenia gravis (MG) being the most common [1–3, 6].

The treatment of TET depends on resectability [1–3]. In unresectable cases, neoadjuvant and/or curative chemo-radiotherapy (chemo-RT) is recommended. For resected cases, adjuvant treatment depends on resection type, Masaoka-Koga stage (s), and WHO classification, which are primary prognostic factors. Additionally, factors such as invasion of surrounding organs, number of invaded organs, tumor size, older age, and performance status have been shown to play a role in survival [1, 7–9]. Masaoka-Koga stage is the pathological staging and is standard for treatment decisions [10]. The prognostic importance of clinical staging based on the tumor-nod-metastasis (TNM) classification developed by the International Association for the Study of Lung Cancer (IASLC) and the International Thymic Malignancy Interest Group (ITMIG) is still under investigation [11].

The decision for postoperative radiotherapy (PORT) in TET varies greatly, with stage and resection type being the most important criteria. In the RYTHMIC study, the distribution of Masaoka-Koga stages I, II, III, and IV were 28%, 41%, 18%, and 12%, while 72%, 4%, 12%, and 12%, for TNM stage (8th edition), respectively [5]. There was a discordance between stages due to the migration from Masaoka-Koga sI -II to TNM sI-II. According to this study, PORT was recommended to only 4% of Masaoka-Koga sI patients, while to 27% of TNM sI patients. Another multicenter study emphasizes that the TNM stage is better at predicting recurrence [12]. The recurrence rates for Masaoka-Koga sII and sIII are 8 and 29%, respectively, and in these cases, significant survival improvement is seen when PORT is used, even with complete resection (R0) [13–15]. Advances in RT techniques have reduced long-term side effects and may further improve survival [3, 16, 17]. In the absence of randomized trials, international working groups have created consensus reports based on data from retrospective and prospective studies to guide diagnosis, treatment, and follow-up [1–3, 5]. Ten-year survival rates reported in the European Society for Medical Oncology (ESMO) guidelines were 84%, 83%, 70%, and 42–53% for Masaoka-Koga sI, sII, sIII, and sIV, respectively, while in the Spanish Society of Medical Oncology (SEOM) guidelines, the rates were 90%, 70%, 55%, and 35% for TNM sI, sII, sIII, and sIV, respectively [1–3].

TC and TNET are less common but are more aggressive histologic types, leading to nodal and distant metastases. A multidisciplinary approach is recommended for treatment. Five-year survival rates for TC and TNET are reported as 55% and 28–75%, respectively [16].

The aim of this study is to evaluate the treatment outcomes and prognostic factors of TET patients treated with RT in our institution in the light of current guidelines.

## Materials and methods

A total of 64 cases diagnosed with TET between January 2000 and December 2023, who received RT ± chemotherapy as decided by a multidisciplinary board, were examined. Written informed consent was obtained from all patients included in the study. The study was conducted in accordance by the Declaration of Helsinki. Local ethical committee approval was granted (2024-14/9).

Staging was performed using thoracic computed tomography (CT) and/or magnetic resonance imaging, and PET/CT also started to be used since in 2006. Surgical evaluation was done in our hospital for 56 patients, whereas 8 patients were evaluated in another hospital and referred to our department for RT. For RT planning, patients were positioned supine with arms elevated for immobilization, and CT simulation images were obtained. The planning target volume was delineated with a 1–1.5 cm margin to include the tumor bed and residual volume. Before June 2008, two-dimensional RT planning was used; from 2008 onwards, three-dimensional conformal RT (3DCRT) and intensity-modulated RT (IMRT) planning were used, and from 2015 onwards, volumetric modulated arc therapy (VMAT) planning was employed. RT was delivered with a linear accelerator to a dose of 45–50 Gy if the surgical margins were negative or close, 54 Gy, if there was microscopic residual disease (R1), or at least 60 Gy if there was macroscopic residual disease (R2) or the tumor was unresectable. Cisplatin-based multi-agent chemotherapy was used for R1‑2 resected and unresectable patients. Toxicity was evaluated according to RTOG criteria. Follow-up included thoracic CT every 6 months for the first 2 years and annually thereafter.

## Statistical analysis

Data were analyzed using SPSS 21 as of February 2024. Overall survival (OS), progression-free survival (PFS), and also cancer-specific survival (CSS) were calculated from the start of RT. Survival was calculated with Kaplan-Meier test and log-rank test was used for group differences. Correlation analysis was done with Spearman’s correlation coefficient for numerical variable relationships, Pearson Chi-square tests, Fisher’s exact test, and Fisher-Freeman-Halton test for categorical variables. Multivariate analysis was performed using the Cox regression model. A *p*-value of ≤ 0.05 was considered as statistically significant.

## Results

Among the patients, 52% were female and the median age was 52 years (range 20–83) (Table 1). At diagnosis, a total of 33% of patients had autoimmune diseases such as MG, autoimmune hepatitis, autoimmune hemolytic anemia, Sjögren syndrome, lupus-like syndrome, and IgA deficiency. Five patients had a history of 6 different primary cancers. The median tumor diameter was 7 cm (range 1.5–20). Comorbidities were present in 67% of patients (*n* = 43), and 14% (*n* = 9) of them had cardiac disorder. Family history of cancer was present in 27% (*n* = 17) of the patients.

**Table 1:** Clinical characteristics of patients

Clinical characteristics	*N* (range/%)
*Age (median, year)*	52 (20–83)
*Male/Female*	31 (48)/33 (52)
*KPS (median)*	90 (70–100)
*Smoking history*	27 (42)
*Smoking pack/year (median)*	20 (5–82.5)
*Histology*	
Thymoma	58 (91)
Thymic carcinoma	4 (6)
Thymic neuroendocrine carcinoma	2 (3)
*Risk group (n: 62)*	
Low risk (A/AB/B1)	25 (40)
High risk (B2/B3/C)	37 (60)
*Tumor size (median, cm)*	7 (1.5–20)
*Myastenia Gravis at diagnosis*	17 (26.5)
*Autoimmune disorder at diagnosis (including MG)*	21 (33)
*Masaoka-Koga stage*	
I	3 (5)
II	39 (61)
III	17 (26)
IV	5 (8)
*TNM stage*	
I	40 (63)
II	7 (11)
III	11 (17)
IV	6 (9)
*SUVmax value (median, n: 21)*	5.5 (1.7–15.8)
*Family cancer history*	17 (27)
*Comorbidity history*	43 (67)

*KPS* Karnofsky performance status, MG Myastenia Gravis

91% of patients were diagnosed with thymoma. Masaoka-Koga and TNM staging ratios for sI, sII, sIII, and sIV were 5%, 61%, 26%, and 8%, and 63%, 11%, 17%, and 9%, respectively. Most of the Masaoka-Koga sII patients (37/39) were classified as TNM sI. Of the Masaoka-Koga sIII patients, 5 were classified as TNM sII and one was classified as TNM sIV.

The WHO types A/AB/B/C and TNET were 5%, 22%, 64%, 6%, and 3%, respectively. Fifteen patients (23%) had indeterminate type B2 and B3 diagnoses. WHO type distribution according to both stages is shown in Table 2.

**Table 2:** Masaoka-Koga and TNM staging related with WHO subclassification

	WHO subclassification									
	A	AB	B1	B2	B3	Unclassified subtype of B (B2-3)	MNT	TC	TNET	Total (*n*, %)
*Masaoka-Koga stage*										
I	1	–	–	1	–	1	**–**	**–**	**–**	*3 (5)*
IIA	1	2	1	1	–	1	–	**–**	1	*7 (11)*
IIB	–	10	4	4	4	6	1	2	1	*32 (50)*
IIIA	–	1	2	2	1	3	**–**	–	**–**	*9 (14)*
IIIB	–	1	–	–	2	3	**–**	2	**–**	*8 (12)*
IVA	1	–	–	1	1	1	**–**	**–**	**–**	*4 (6)*
IVB	–	–	–	–	1	–	**–**	**–**	**–**	*1 (2)*
*Total (n, %)*	*3 (5)*	*14 (22)*	*7 (11)*	*9 (14)*	*9 (14)*	*15 (23)*	*1 (2)*	*4 (6)*	*2 (3)*	*64 (100)*
*TNM stage*										
I	2	12	4	6	4	8	1	1	2	*40 (63)*
II	–	–	2	1	1	2	–	1	–	*7 (11)*
IIIA	–	2	1	1	–	4	–	1	–	*9 (14)*
IIIB	–	–	–	–	2	–	–	–	–	*2 (3)*
IVA	1	–	–	1	1	1	–	–	–	*4 (6)*
IVB	–	–	–	–	1	–	–	1	–	*2 (3)*
*Total (n, %)*	*3 (5)*	*14 (22)*	*7 (11)*	*9 (14)*	*9 (14)*	*15 (23)*	*1 (2)*	*4 (6)*	*2 (3)*	*64 (100)*

MNT multinodular tymoma, TC thymic carcinoma, TNET thymic neuroendocrin tumor

Treatment characteristics are summarized in Table 3. Before RT, 81% of patients had R0 resections. In 24 patients (37.5%), at least one surrounding organ invasion was observed, with pericardial involvement (22%) being the most common. The median interval between surgery and RT was 49 days (range 15–95). Patients received a median total dose of 5040 cGy (range 1620–6596) over a median time of 42 days (range 11–56). Three patients with Masaoka-Koga sI received PORT due to a high mitotic/proliferative index or WHO type B2‑3. Sixteen patients (25%) received a median of 5 cycles (range 1–7) of chemotherapy, either neoadjuvant, concurrent, or adjuvant. Dosimetric data were available for 30 patients treated after 2015, and all were within tolerance limits.

**Table 3:** Treatment characteristics of patients

Treatment characteristics	*N* (range/%)
*Resection type (before RT)*	
R0	52 (81)
R1	4 (6)
R2	3 (5)
Biopsy	5 (8)
*Lymph node dissection*	28 (44)
Number of removed lymph nodes (median)	3 (1–13)
*Surrounding organ invasion*	24 (37.5)
Single organ	10 (16)
2 organs	6 (9)
≥ 3 organs	8 (12.5)
*Involved organs*	
Pericardium	14 (22)
Pleura	12 (19)
Lung	10 (16)
Great vessels	10 (16)
Chest wall	4 (6)
Phrenic nerve	1 (2)
*Surgery-RT interval (median, days)*	49 (15–95)
*RT dose (median, cGy)*	5040 (1620–6596)
*Fraction dose (median, cGy)*	180 (180–200)
*RT time (median, days)*	42 (11–56)
*Organ at risk (n: 30)*	–
Mean lung dose (Gy)	8.40 (5.17–18.99)
Lung V5 (%)	41.48 (25.55–72.7)
Lung V20 (%)	15.15 (4.00–40.99)
Mean heart dose (Gy)	7.81 (0.73–24.81)
Heart V5 (%)	30.92 (0.00–84.22)
Heart V30 (%)	8.77 (0.00–42.55)
Mean esophagus dose (Gy)	11.24 (6.27–25.80)
Esophagus V50 (%)	0.00 (0.00–1.38)
Mean spinal cord dose (Gy)	5.08 (2.43–10.92)
Maxium spinal cord dose (Gy)	21.25 (3.33–44.01)
*Chemotherapy history*	16 (25)
Chemotherapy cycles (median)	5 (1–7)

Before RT, surgery was performed on 2 patients who had received neoadjuvant chemotherapy. One of them had R0 resection and showed a pathological complete response while other patients underwent R2 resection. A total of 59 patients underwent surgery before RT. Surgery was performed on other 2 patients who had received neoadjuvant RT or chemoRT. One of them had R0 resection and showed a pathological complete response while other patient underwent R2 resection. One biopsy-only patient was lost to follow-up after 3 months, having received 1620 cGy of RT and 1 cycle of chemotherapy. One R0 resected patient with MG, on pyridostigmin bromide and prednisolone, received 4140 cGy of RT, and died of pneumonia. Two R0 resected patients developed atypical pneumonia during RT and could receive 2520 and 4600 cGy of RT. In total, 45 patients (70%) experienced grade 1–4 acute side effects during RT. Grade 2 and over esophagitis and hematologic toxicity were observed in 17% and 9% of patients, respectively. Acute radiation pneumonia was observed in a total of 9% of patients in a median time of 1 month (range 1–3).

The cut-off date for follow-up was February 01, 2024, and the median follow-up was 70 months (range 1.5–268). Five second primary cancers developed in five patients over a median period of 31 months (range 6–123), one of whom had a previous history of cancer. A total of 11  primary cancers were found in 9 patients (14%) with a median age of 61 (range 32–71) years at diagnosis. During follow-up, 5 patients developed autoimmune diseases at a median time of 3 years (range 2–7), with a total of 26 patients (40%) having autoimmune diseases. One patient developed lung tuberculosis at 11 months after RT is alive with a follow-up of 237 months. After RT, 11 patients (17%) developed new cardiac disorder, one of whom had cardiac morbidity at diagnosis. One of the two patients who died of cardiac causes had local recurrence and these cases were treated before 2015. Two patients had a history of multinodular goiter at the time of diagnosis. During follow-up, new multinodular goiter developed in 11 (17%) patients, and 2 of them were diagnosed with thyroid cancer.

A total of 15 patients (23%) experienced recurrence at a median time of 30 months (range 6.5–106), involving the anterior mediastinum, pericardium, pleura, and bone. 20% of recurrent patients occurred more than 5 years later. Fourteen patients (22%) had intrathoracic recurrence, of which 11 (17%) were only local recurrence, and bone metastases were also observed in 2 patients (3%, one with intrathoracic recurrence). The 5‑, and 10-year local control (LC) rates were 82.4%, and 74.3%, and the 5‑, and 10-year intrathoracic control rates were 78.9%, and 67%, respectively. The 5‑year LC rates were significantly different between Masaoka-Koga sI-II and sIII-IV (94.8% vs 56.9%, *p* = 0.013), but not for TNM sI-II and sIII-IV (89.6% vs 59.3%, *p* = 0.070). Patients with recurrence underwent surgery, chemotherapy or RT. Second series RT was administered to 6 patients at a median dose of 5040 cGy (range 3960–6000). Three patients with recurrence were alive after 34, 106, and 126 months of follow-up.

During the follow-up period, 28 patients died. Causes of death included progression (*n* = 12), surgical complication after progression (*n* = 2), second primary cancers (*n* = 4), myasthenic crisis/immunodeficiency (*n* = 3), cardiac causes (*n* = 2), radiation pneumonitis during RT (*n* = 1), recurrent pneumonia (*n* = 1), and multiple organ failure (*n* = 1). The cause of death was unknown in 2 patients with R0 resection with 3.5 and 33 months follow-up. Mortality rates due to progression or other causes were 22% (14/64) each.

The mean OS for TC was 44 months. In these patients, Masaoka-Koga and TNM stage showed discordance due to clinical N2 lymph node involvement. Only one R0 resected TC patient was alive with 21 month follow-up. Two TNET patients died due to second primary cancer or cardiac reason with 119.5 and 174.5 months follow-up, respectively.

**Fig. 1:**
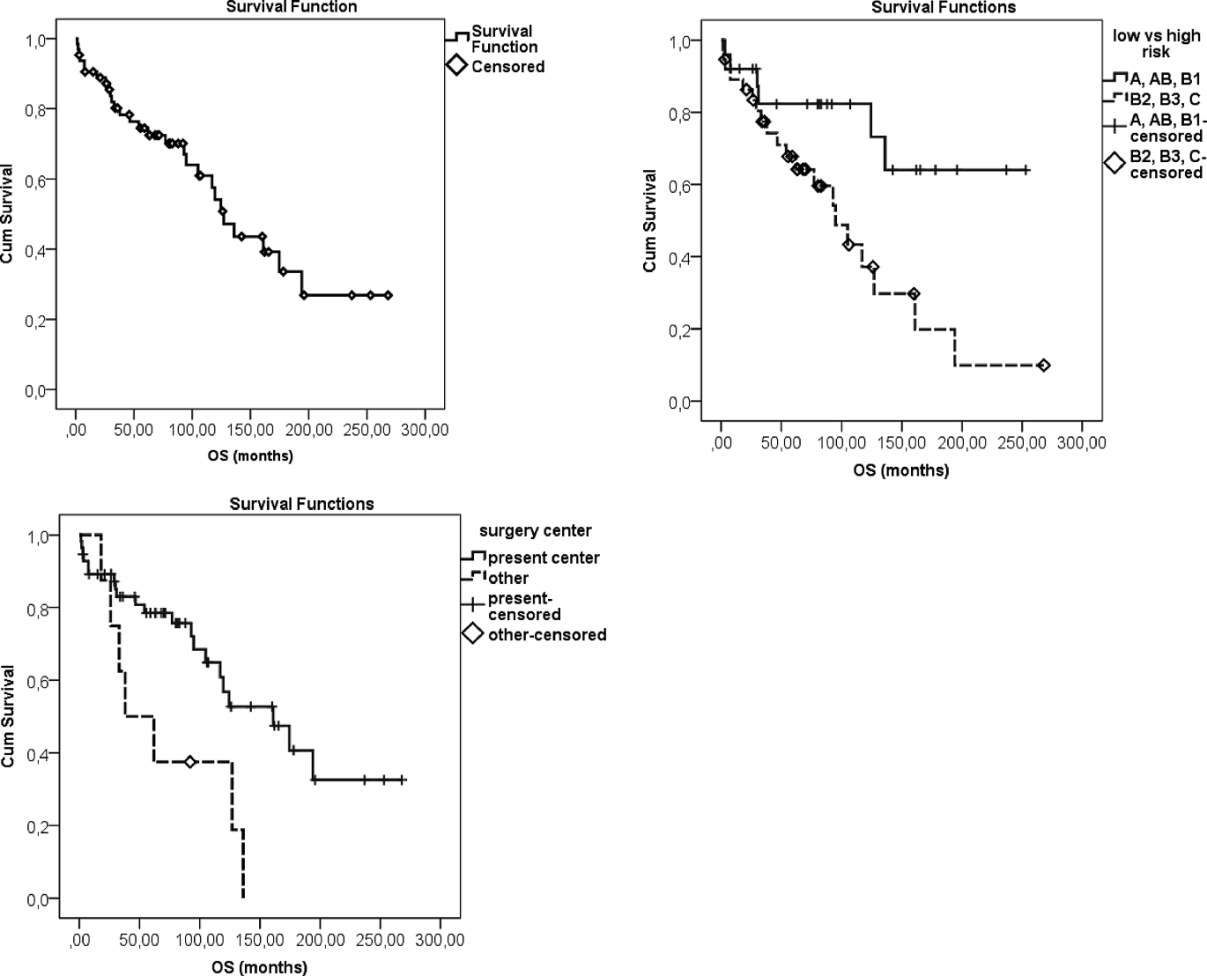
Overall survival (OS) and prognostic factors

**Fig. 2:**
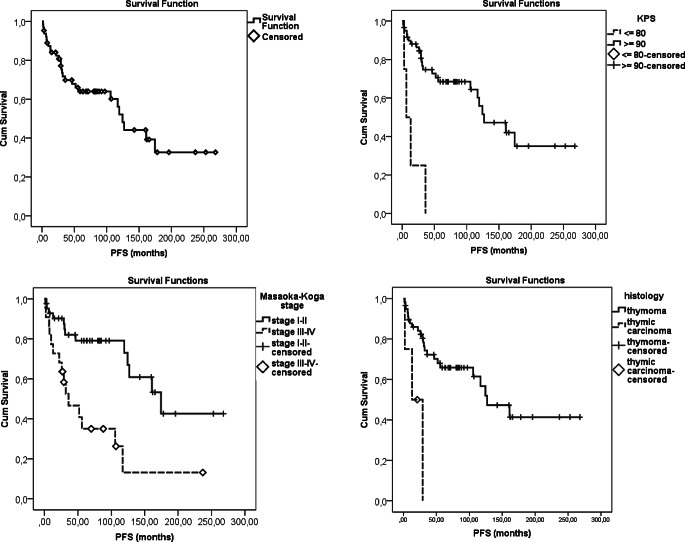
Progression-free survival (PFS) and prognostic factors

The mean and 5‑year OS and PFS were 141 months, and 74.5%, and 138 months and 63.9%, respectively (Table 4). The mean and 5‑year CSS was 188 months and 88.4%, respectively. There was no difference in survival in terms of RT technique or year of treatment. Univariate analysis identified Karnofsky performance status (KPS), histology, risk group (B2/B3/C), Masaoka-Koga stage, TNM stage, resection type, surrounding organ involvement, and surgical center as significant prognostic factors in terms of all survival types (i.e., OS, PFS, CSS) (*p* < 0.005). Advanced age (*p* = 0.022) was an unfavorable factor only for OS. In multivariate analysis, high risk and another surgery center for OS (*p* < 0.005) and KPS ≤ 80, TC and Masaoka-Koga sIII-IV for PFS and CSS (*p* < 0.001) were significant as unfavorable prognostic factors (Table 4; Figs. 1 and 2). Correlation analysis showed a linear correlation between Masaoka-Koga and TNM stages (*p* < 0.001, r = 0.923).

**Table 4:** Univariate and multivariate analysis for survival

Prognostic factors	OS months (mean ± SD)	*P* value	PFS months (mean ± SD)	*P* value	CSS months (mean ± SD)	*P* value
**–**	141 (± 14)	**–**	138 (± 16)	**–**	188 (± 17)	**–**
	5‑year 74.5%		5‑year 63.9%		5‑year 88.4%	
	10-year 54.2%		10-year 52.1%		10-year 72.4%	
*Age (n)*	–	*0.022*	–	0.108	–	0.333
≤ 59 (41)	165 (± 18)		161 (± 20)		196 (± 18)	
≥ 60 (23)	89 (± 14)		89 (± 15)		131 (± 17)	
*KPS (n)*	*–*	*<* *0.001*	*–*	*<* *0.001*	*–*	*<* *0.001*
≥ 90 (60)	150 (± 15)		147 (± 16)		198 (± 17)	
≤ 80 (4)	27 (± 13)		14 (± 7)		35 (± 15)	
*WHO type (n)*	*–*	*0.011*	*–*	*0.003*	*–*	*<* *0.001*
Thymoma (58)	150 (± 16)		150 (± 17)		221 (± 18)	
Thymic carcinoma (4)	44 (± 15)		18 (± 7)		99 (± 22)	
*WHO subgroup (n)*	*–*	*0.011*	*–*	*0.008*	*–*	*0.039*
Low risk (25)	189 (± 21)		187 (± 22)		216 (± 18)	
High risk (37)	107 (± 16)		99 (± 20)		154 (± 23)	
*Masaoka-Koga stage (n)*	*–*	*0.006*	*–*	*<* *0.001*	*–*	*<* *0.001*
I‑II (42)	173 (± 19)		172 (± 19)		243 (± 13)	
III-IV (22)	92 (± 16)		71 (± 19)		113 (± 19)	
*TNM stage (n)*	*–*	*0.003*	*–*	*0.001*	*–*	*0.004*
I‑II (47)	163 (± 17)		163 (± 19)		163 (± 19)	
III-IV (17)	81 (± 17)		65 (± 20)		74 (± 18)	
*Resection type (n)*	*–*	*0.015*	*–*	*0.003*	*–*	*<* *0.001*
R0 (52)	153 (± 16)		152 (± 18)		214 (± 20)	
R + (12)	81 (± 21)		57 (± 23)		88 (± 22)	
*Invasive structure(n)*	*–*	*0.035*	*–*	*0.004*	*–*	*0.001*
Absent (40)	166 (± 20)		165 (± 20)		241 (± 14)	
Present (24)	102 (± 17)		84 (± 20)		124 (± 20)	
*Surgery Center (n)*	–	*0.011*	–	*0.013*	*–*	*0.001*
Present center (56)	155 (± 16)		154 (± 17)		205 (± 17)	
Another center (8)	71 (± 18)		58 (± 17)		81 (± 20)	
*Cox regression analysis*	*HR (95% CI)*	*P value*	*HR (95% CI)*	*P value*	*HR (95% CI)*	*P value*
*KPS* *≤* *80*	–	**–**	4.6 (1.5–14.4)	*0.008*	13.8 (2.6–73.5)	*0.002*
*Thymic carcinoma*	–	**–**	3.8 (1.0–14.3)	*0.043*	9.4 (1.8–49.3)	*0.007*
*Masaoka-Koga sIII-IV*	–	**–**	3.7 (1.6–8.3)	*0.001*	14.0 (2.9–66.2)	*0.001*
*High-risk group*	33.2 (2.8–386.8)	*0.005*	–	–	–	**–**
*Another surgery center*	11.6 (2.0–67.6)	*0.006*	–	–	–	**–**

OS overall survival, PFS progression-free survival, CSS cancer-specific survival, KPS Karnofsky performance status, HR hazard ratio, CI confidence interval

## Discussion

Thymic epithelial tumors are rare, and the primary treatment is surgery [1–3]. Decisions regarding adjuvant therapy depend on the type of resection, Masaoka-Koga stage, and histological classification, with no complete consensus available. Recurrence in R0 resected TET has been reported at a rate of 10–15%, typically occurring within an average of 5 years (range 3–7) [1]. Several predictive and prognostic factor-based classifications have been developed to estimate recurrence, and international guidelines have been established since 2015 [1–3, 5, 8, 12]. This study involves an analysis of single-center TET patient outcomes in the light of current guidelines.

According to a Japanese study, the incidences of thymoma, TC, and TNET were 88%, 10%, and 2% respectively, while the IASLC staging committee reports these incidences as 84%, 15.5%, and 1.5% based on data from 11,347 patients [2, 18]. In the prospective RYTHMIC study, the frequency of occurrence for WHO types A, AB, B, C, and others are reported as 10%, 25%, 53%, 11%, and 1% respectively [5]. However, the difficulty in distinguishing between WHO B2 and B3 types is also noted [19].

The role of PORT in R0 resected Masaoka-Koga sI cases has not been demonstrated and is not recommended [1, 20]. On the other hand, the role of PORT in R0 resected sII cases is debated. The ITMIG study reported a survival benefit with PORT in R0 resected Masaoka-Koga sII cases [13]. In a study by Jackson et al. involving 4056 cases using 3DCRT/IMRT techniques, no benefit was shown in Masaoka-Koga sIIa, whereas survival benefit was reported in sIIb-III and incompletely resected cases [21]. Conversely, in the SEER analysis, no difference in OS and CSS was found with PORT in Masaoka-Koga sIIb cases [22]. The WHO classification is reported as an independent prognostic factor with A/AB/B1 indicating low risk and B2/B3/TC indicating high risk [23]. In Lee et al.’s study of R0 resected 406 cases, 5‑year freedom from recurrence was reported as 99.3% for Masaoka-Koga sI and A/AB/B1 cases, emphasizing that adjuvant RT is unnecessary [20]. The Italian study group suggests PORT for sIIb and B2-3 cases, whereas the ESMO guidelines recommend PORT for sIIb or sIIa and B2-3 cases [1, 24].

Masaoka-Koga staging is standard for treatment decisions. The TNM staging system was developed to address the need for a universal and consistent classification [11]. The 8th edition of TNM staging can be adapted to all histological types, with incidences reported as 81.4% for sI, 2.7% for sII, 10.5% for sIII, and 5.2% for sIV, and recurrence rates of 5%, 18%, 32%, and 46%, respectively. In the RYTHMIC study, most patients showed stage migration from Masaoka-Koga sII to TNM sI [5]. In their study, PORT was recommended for 4% of Masaoka-Koga sI patients, and 27% of TNM sI patients, with the multidisciplinary board decision aligning 92% with the ESMO recommendations. Factors considered for PORT included TC/B3 histology, Masaoka-Koga stage, TNM stage, and incomplete resection, while performance status, presence of comorbidities, and stage discordance were taken into consideration in cases where PORT was not recommended. In a recent study of R0 resected 445 cases, TNM staging was highlighted as better predictive of recurrence than Masaoka-Koga staging [12].

Patients with TC and TNET are often at advanced stages and have a worse prognosis compared with thymoma [16]. In a study by Rimner et al., involving 462 TC cases, survival benefit with PORT was reported in incompletely resected or sIII-IV cases [25]. SEER analysis reported that Masaoka-Koga stage, type of resection, lymph node or distant metastasis, tumor size, and receiving RT are predictive of survival [26]. The aggressive nature and high recurrence rates of TNET cases suggest that adjuvant RT and chemotherapy are necessary even for R0 resected cases [16].

In our study, the incidence rates of thymoma, TC, and TNET were 91%, 6%, and 3% respectively, consistent with published data. 95% of our Masaoka-Koga sII cases were classified as TNM sI. The low incidence of Masaoka-Koga sI cases in our study (5%) is due to fact that they were not referred to our department because there was no indication for PORT. Due to high risk, only 3 Masaoka-Koga sI cases underwent PORT. 5‑year OS for Masaoka-Koga sI-II and sIII-IV were 81.6% and 61%, and for TNM sI-II and sIII-IV were 81.6% and 54.1%, showing compatibility (*p* = 0.001, r = 0.881).

In TET patients, older age, larger tumor size, and the presence of MG are also reported as independent poor prognostic factors [6, 23]. On the other hand, MG was associated with favorable features (i.e. earlier stage, complete resection status) in many studies [2, 6]. Significant threshold values for tumor size are reported as 5 to 10 cm in different studies [1, 27]. Lopez et al. reported that poor performance status was associated with worse survival in 192 patients who received PORT and were followed for 10 years [9]. Recent studies emphasize that organ invasion indirectly reflects advanced stage and poor prognosis [8, 15]. Funaki et al. reported 35% organ invasion in R0 resected 306 thymoma cases, with single organ, two organs, and three or more organs involvement rates of 13.7%, 16.5%, and 14.1% respectively [8]. We identified older age and poor performance status were significant prognostic factors in terms of OS demonstrating that prognosis is influenced not only by tumor-related factors but also by patient-related factors. We did not identify significant difference for presence of MG because of overlap with other prognostic factors. A total of 37.5% organ invasion was seen in our study, with rates of 1, 2, and ≥ 3 organs involvement were 16%, 9%, and 12.5%, respectively, consistent with the literature. The presence of organ invasion was significant for all survival groups in univariate analysis.

The first site of recurrence in TET is often the pleura, pericardium, or lung, with predictive factors for recurrence including advanced stage, large tumor size, non-thymoma histology, and incomplete resection [9, 20, 28]. Lopez et al. reported 38% locoregional recurrence and 16% distant recurrence in 192 cases receiving PORT, with 22% of local recurrences being in-field recurrences [9]. In our study, a total of 23% recurrences occurred, 17% of which were within the field. The 5‑year LC was 82.4%, whereas the 5‑year intrathoracic control was 78.9%. While there was a significant relationship between LC and Masaoka-Koga stage, no significant relationship was found between LC and TNM stage, and this was attributed to stage migration.

The frequency of MG in thymoma patients is reported as 30%, often associated with early diagnosis and good prognosis [1, 6, 28]. In contrast, other autoimmune diseases occur in 10% of cases and have a worse prognosis. Opportunistic respiratory infections are common in these patients [29]. Additionally, since TET patients are relatively young and may live longer, treatment-related side effects must be considered. High death rates due to lung and heart toxicity in TET patients highlight the importance of RT dose and technique [1, 5, 13]. Gomez et al. reported dose-volume threshold values for organ at risk in patients with TET [30]. Current guidelines emphasize starting adjuvant RT within 3 months, using at least 3DCRT/IMRT techniques, and selecting the RT dose based on the type of resection [1–3]. The Delphi consensus report recommends 4DCT simulation at 82% and VMAT technique at 88% [17]. It is emphasized that secondary primary cancers are seen in 17–28% of TET patients at diagnosis and follow-up, with the age of diagnosis being crucial rather than treatment [28]. In our study, 33% of patients had autoimmune diseases at diagnosis, which increased to 40% during follow-up, consistent with literature. RT was initiated within 3 months in all but one patient who received treatment on the 95th day after biopsy. Patients received a median of 5040 cGy RT. Dose constraints were not exceeded in any patients. Among patients treated before 2015, two patients died of pneumonia who had also MG, and other two patients died of cardiac disorder during follow-up. These patients received between 4140 to 6000 cGy RT and the doses to organs at risk were unknown. After 2015, 3 out of 30 patients developed new cardiac disorder, all were alive at the last follow-up, and no relationship was found in terms of heart doses. There was no survival difference based on RT technique or year of treatment. Second primary cancers were present in a total of 14% of cases, and their median age at diagnosis was 61 years, consistent with literature.

Advances in surgical techniques have reduced perioperative complications and improved survival, and also the skills and experiences of the surgeon are important [31]. PFS and CSS were significantly better in those who underwent surgery in our center.

Advancements in the diagnosis, staging, and treatment of TET continue. A recent development is the use of radiomics for preoperative differentiation of low or high risk with a reported accuracy of 92%, sensitivity of 96%, and specificity of 90% [32]. The genetic profile of TET cases is heterogeneous, and research is ongoing into the use of targeted agents and immunotherapy in systemic treatment [1–3]. Studies including new prognostic factor-based TNM staging (9th edition) are underway [18, 27]. Mediastinal pleural invasion is a pathological finding that is not easily recognized and is difficult to detect in clinical staging, suggesting its exclusion from staging. Furthermore, because survival differences have been demonstrated, it is planned to include tumor size with a 5 cm threshold in staging, and to place lung and phrenic nerve invasion in T2 rather than T3. Despite the shown relationship between nodal staging and survival, issues with pathological and clinical nodal staging discrepancies and the significance of the number of metastases remain unresolved.

The limitations of the study are the inclusion of patients with TC, TNET and unresectable patients, and the heterogeneity in RT techniques. Strengths of the study include the long follow-up period and demonstration of the importance of current prognostic factors.

In conclusion, in this single-center study of 64 TET patients treated with RT, a 5-year LC rate of 82.4% was achieved with a follow-up of 70 months. 20% of recurrences occurred after 5 years and long follow-up is required. In univariate analysis, the presence of invasive organs and TNM stages were identified as significant new prognostic factors for survival. In multivariate analysis, the prognostic significance of KPS, histology, Masaoka-Koga stage, risk group, and surgical center experience was confirmed. Advances in the diagnosis, staging, and treatment of TET will enable more personalized treatment approaches.

## Data Availability

All data generated or analyzed during this study are included in this published article
